# Usefulness of a standardized scleral buckling technique for primary rhegmatogenous retinal detachment

**DOI:** 10.1371/journal.pone.0323881

**Published:** 2025-05-15

**Authors:** Hisanori Imai, Maya Kishi, Yasuyuki Sotani, Hiroko Yamada, Wataru Matsumiya, Akiko Miki, Sentaro Kusuhara, Makoto Nakamura

**Affiliations:** 1 Department of Surgery, Division of Ophthalmology, Kobe University Graduate School of Medicine, Kobe, Japan; 2 Department of Ophthalmology, Kansai Medical University, Hirakata, Japan; Akita University: Akita Daigaku, JAPAN

## Abstract

**Purpose:**

Rhegmatogenous retinal detachment (RRD) requires timely treatment to prevent vision loss. Buckling surgery is a vital intervention; however, surgeon proficiency in buckling surgery is declining, and efforts are needed to preserve it. This study evaluated the efficacy of a standardized buckling surgical technique for primary RRD.

**Methods:**

In total, 69 eyes were included. The technique involved placing #287 silicone tires in the retinal break regions with a 9–10-mm mattress suture positioned using the posterior edge of the rectus muscle insertion as a reference point. Circumferential buckling was performed using a silicone band (#240) and silicone sleeve (#270).

**Results:**

The 6-month follow-up showed an initial anatomical success rate of 92.8%, which reached 100% at the final follow-up. The mean best-corrected visual acuity was 0.16 ± 0.45 pre-operation and 0.05 ± 0.24 at the 6-month follow-up (p = 0.05).

**Conclusion:**

This technique utilizes the characteristics of the anatomical positional relationships between the scleral insertion of the lateral rectus muscle, which is reported to overlap with the vitreous base insertion, and the ora serrata and shows promise in identifying retinal breaks and determining buckling positions, indicating its potential effectiveness for the treatment of RRD. The favorable anatomical and visual outcomes support the potential usefulness of this technique in buckling surgeries.

## Introduction

Rhegmatogenous retinal detachment (RRD) is an emergency ocular condition that can lead to blindness if left untreated [1]. Several treatment options such as pars plana vitrectomy (PPV), pneumatic retinopexy, and buckling have been reported to yield favorable outcomes [2]. Among these options, buckling surgery is considered an important procedure and is particularly suitable for young individuals with atrophic holes and minimal vitreous liquefaction [3].

The number of buckling surgeries performed worldwide has been decreasing in recent years [4]. This can be attributed to advancements in PPV, which has become increasingly less invasive and has expanded indications [5]. Additionally, there are long-standing issues associated with buckling surgery [4]. Specifically, conventional techniques using an indirect ophthalmoscope for fundus observation have limitations such as a narrow surgical field that makes it difficult to detect small retinal tears. Moreover, the lack of shared surgical field visualization between the surgeon and assistant makes it challenging to educate and train on certain aspects such as the strength of retinal cryopexy and the height and position of the buckling material. In addition, despite the need for significant experience to acquire proficiency in buckling surgery, there has been a relative decrease in the number of procedures. Consequently, this trend may contribute to an increasing number of ophthalmologists who are unable to attain full proficiency in the surgical buckling technique. Furthermore, the impact of these issues has led many surgeons to prefer PPV over buckling surgery, further exacerbating the decline in the number of buckling surgeries performed. Consequently, the decrease in the number of surgeons proficient in buckling surgery has become a significant concern, necessitating discussions on how to ensure the continuation and inheritance of buckling surgery.

Recently, a buckling surgery technique utilizing chandelier illumination and a wide-angle fundus-viewing system demonstrated favorable surgical outcomes [6–9]. Despite some concerns regarding associated complications, this technique offers several advantages such as shared surgical field visualization for the surgeon and assistant, facilitation of education, and improved detection of small retinal tears under wide-angle fundus observation [6–9]. Consequently, this technique has partially addressed the aforementioned issues associated with buckling surgery, making it a more reliable procedure. However, determining the position of the buckling material for retinal tears still relies heavily on the subjective observation and experience of the surgeon, akin to that in conventional techniques using an indirect ophthalmoscope. Consequently, beginners may encounter difficulties in accurately determining the position of the buckling material. In recent years, the development of intraoperative optical coherence tomography has enabled real-time confirmation of the relationship between the position of the buckling material and the location of retinal tears [10–13]. This advancement is expected to provide a solution for achieving accurate positioning of buckling materials and is likely to be widely adopted. However, the limited availability of intraoperative optical coherence tomography may hinder its widespread use. Hence, ongoing discussions are warranted to address the inheritance and propagation of the buckling technique while effectively addressing the challenges associated with buckling surgery.

Herein, we report the development of a standardized buckling technique that ensures consistent positioning of buckling material and achieves favorable postoperative outcomes.

## Materials and methods

The study population included 69 eyes of 66 patients who underwent standardized buckling surgery for primary RRD at our institution between January 2014 and March 2022. A retrospective analysis of the medical records was conducted. The study was conducted in accordance with the Declaration of Helsinki and was approved by the Ethics Committee of Kobe University Graduate School of Medicine (No. B220037, May 26, 2022). We accessed the medical records on June 5, 2023, for research purposes and had access to information that could identify individual participants during or after data collection. The requirement for informed consent was waived by the committee because of the retrospective observational design of the study. Nonetheless, patients could opt out and withdraw consent at any time through the hospital homepage. Patients with retinal detachment associated with an ora serrata break, those with proliferative vitreoretinopathy (grade C), those with an unknown tear location before surgery, those with vitreous hemorrhage affecting fundus visibility, and those who underwent concomitant vitreous surgery were excluded.

The following variables were extracted from medical records and used for statistical analysis: sex, age, number and location of retinal tears, extent of retinal detachment, presence of foveal detachment, operation time, primary anatomical success, final anatomical success, occurrence of intraoperative and postoperative complications, preoperative visual acuity, visual acuity at 6 months after surgery, preoperative refractive error, refractive error at 6 months after surgery, preoperative intraocular pressure (IOP), and IOP at 6 months after surgery.

### Surgical procedure

**Fig 1:**
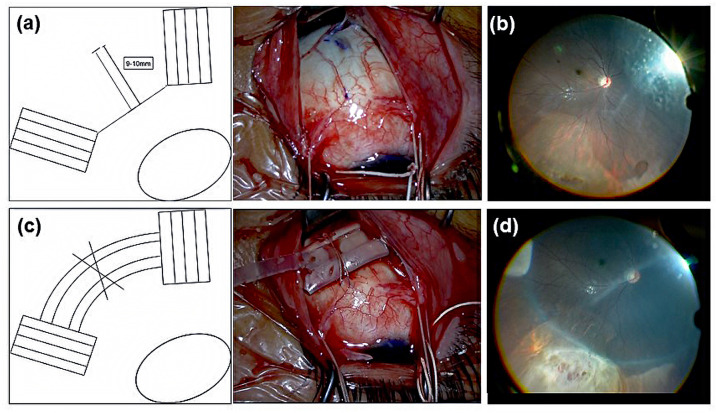
Surgical procedure for the novel scleral buckling technique. (a) Using the posterior edge of the insertion of the rectus muscle as a reference point, mattress sutures (9–10 mm) with 5-0 Dacron are placed between the rectus muscles of the quadrant containing the retinal tear and adjacent quadrants, ensuring that the tear site remains at the center. (b) Fundus image before scleral buckling. Rhegmatogenous retinal detachment with subretinal fluid and attached macula are observed. (c) A silicone tire (#287) is placed over the previously sutured mattress area. Circumferential buckling is performed using a silicone band (#240) and sleeve (#270). (d) Fundus image after scleral buckling. Under a microscope, any unexpected complications in the fundus are checked, and the position of the buckling material is confirmed.

All surgeries were performed under general anesthesia to prevent patient discomfort. After induction of general anesthesia, all surgical manipulations were performed under a surgical microscope. Following the conjunctival incision along the limbus, Tenon’s capsule was exposed, and four lateral rectus muscles were identified. Traction sutures were placed beneath the lateral rectus muscle. Subsequently, using the posterior edge of the rectus muscle insertion as a reference point, 5–0 Dacron mattress sutures (9–10 mm) (Alcon Laboratories, Inc., Fort Worth, TX, USA) were placed between the rectus muscles of the quadrant containing the retinal tear and adjacent quadrants, ensuring that the tear site remained at the center (Fig 1a). For example, if the tear was located at the 7 o’clock position, the mattress sutures were placed at the 4:30, 7:30, and 10:30 positions. In other quadrants, a half-thickness scleral incision centered 6 mm posterior to the posterior edge of the rectus muscle insertion was created using a crescent knife (MCU26 Crescent Bevel Up 2.3 mm; Mani Inc., Tochigi, Japan) to secure a silicone band. Subsequently, a 25-gauge trocar cannula (25GA Valved Entry System; Alcon Laboratories) with a closure valve was placed 4 mm posterior to the limbus. The trocar cannula’s placement was adjusted according to the location of the retinal tear, and it was typically positioned opposite to the causative tear. For example, if the causative tear was located at the 3 o’clock position, the trocar cannula was placed as close as possible to the 9 o’clock position. Similarly, if multiple retinal tears were present in the inferior quadrant, the trocar cannula was placed at the 12 o’clock position.

A Resight system (Resight®; Carl Zeiss Meditec, Jena, Germany) with 25-G chandelier illumination (Chandelier lighting system; Alcon Laboratories) was placed into the cannula and turned on. Fundus observation was then performed, and after identifying the location of the causative tear (Fig 1b), transscleral subretinal fluid drainage was performed under a microscope in all cases. The drainage site was sutured using 8–0 Vicryl (Ethicon Inc., Raritan, NJ, USA). Subsequently, cryotherapy was applied to the causative tear. A silicone tire (#287, MIRA Inc., Waltham, MA, USA) was placed over the previously sutured mattress area, and circumferential buckling was performed using a silicone band (#240, MIRA Inc.) and silicone sleeve (#270, MIRA Inc.) (Fig 1c). The chandelier light was then turned on again, any unexpected complications in the fundus were checked under microscopic observation, and the position of the buckling material was confirmed (Fig 1d). The cannula was then removed, and vitreous incarceration into the scleral wound was confirmed. If necessary, the incarcerated vitreous was excised, and the wound was sutured using 8–0 Vicryl. The conjunctiva was also sutured using 8–0 Vicryl, and the surgery was concluded. S1 Video provides an overview of this novel surgical technique.

### Statistical methods

For all variables, the mean values and standard deviations are reported. Best-corrected visual acuity (BCVA) was converted to the logarithm of the minimum angle of resolution for statistical analysis. Paired t-tests were conducted to assess changes in BCVA, refractive error, and IOP. Statistical analyses were performed using SPSS software (version 24.0; IBM Corporation, Armonk, NY, USA). Statistical significance was set at *P* < 0.05.

## Results

Perioperative demographic data of the patients are listed in Table 1. The primary anatomical success rate was 92.8% (64/69 eyes). PPV was performed for eyes that did not achieve initial reattachment, resulting in final anatomical success in all cases. No significant intraoperative complications were observed. The postoperative complications are shown in Table 2. High postoperative IOP occurred in 6 of 69 eyes, although it was controlled with topical medication. Cataract progression was observed in 2 of 69 eyes; this was addressed by successful cataract surgery, leading to improvement in visual acuity. Buckle infection occurred in 1 of 69 eyes, and the buckle was removed 3 months after the initial surgery; however, the retinal reattachment remained intact after removal. Postoperative macular edema occurred in 1 of 69 eyes and improved with a sub-tenon injection of triamcinolone acetonide. A full-thickness macular hole was observed in 1 of 69 eyes, although it was successfully closed using a 27-gauge vitrectomy combined with an inverted internal limiting membrane flap technique.

**Table 1:** Perioperative demographic data of the patients.

Characteristics	
Number of eyes	69
Sex, men/women	45/24
Age (years), mean±SD	28.9 ± 10.7
Number of breaks, mean±SD	1.88 ± 1.33
Location of breaks, superior/ inferior	39/30
Quadrant of retinal detachment, 1/2/3/4	19/35/9/6
Foveal detachment, macular on/macular off	37/32
Mean operative time (min), mean±SD	125.76 ± 49.22
Initial anatomical success	64 (92.8%)
Final anatomical success	69 (100%)

Abbreviations: SD, standard deviation.

**Table 2:** Postoperative complications.

Complication	n (%)
Elevated intraocular pressure	6 (8.7%)
Progression of cataract	2 (2.9%)
Buckle infection	1 (1.4%)
Macular edema	1 (1.4%)
Full-thickness macular hole	1 (1.4%)

The mean preoperative BCVA was 0.16 ± 0.45. At the 6-month follow-up, the mean BCVA was 0.05 ± 0.24, and no significant difference from the preoperative BCVA was observed (*P* = 0.05). The mean preoperative refractive error was −5.71 ± 4.13 diopters (D). At the 6-month follow-up, the mean refractive error was −8.84 ± 3.59 D, which was significantly different from the preoperative value (*P* < 0.001). The mean preoperative IOP was 14.8 ± 3.6 mmHg, and the mean IOP at the 6-month follow-up was 14.9 ± 3.1 mmHg, showing no significant change during the observation period (*P* = 0.41) (Table 3).

**Table 3:** Perioperative change in each parameter.

	Before surgery	6 months	*P*
BCVA (logMAR)	0.16 ± 0.45	0.05 ± 0.24	0.05
Refractive error (D)	−5.71 ± 4.13	−8.84 ± 3.59	<0.001
IOP (mmHg)	14.9 ± 3.6	14.9 ± 3.1	0.41

Abbreviations: BCVA, best-corrected visual acuity; D, diopters; logMAR, logarithm of the minimum angle of resolution; IOP, intraocular pressure.

## Discussion

In this study, we investigated the utility of a standardized buckling technique for primary RRD. Consequently, we achieved a good initial reattachment rate of 92.8% (64/69 eyes).

In buckling surgery, proper placement of the buckling material is crucial. Inaccurate positioning of the buckling material can lead to a failure in retinal break closure and increase the risk of retinal redetachment [14]. However, even with conventional techniques using an indirect ophthalmoscope or a combined approach with chandelier illumination and a wide-angle fundus-viewing system, accurate observation of the retinal break and position of the buckling material from the anterior (corneal) side by the surgeon is necessary. Depending on the presence of residual subretinal fluid or the height of the buckling protrusion, it can be challenging to accurately determine the relationship between the retinal break and the position of the buckling material.

The successful mechanism of buckling surgery for RRD involves alleviating the inertial force caused by the movement of the vitreous body during eye movement, known as vitreous traction, by placing the buckling material on the retinal break. This helps achieve primary closure of the break, and secondary closure is achieved through cryotherapy-induced retinal adhesions. In cases with a flap tear retinal break, accurate placement of the buckling material involves selecting an appropriately sized buckling material and precisely placing it to exert the necessary force on both the strong vitreous traction area around the peripheral side of the break and the entire break area. In cases with an atrophic hole, selecting an appropriately sized buckling material capable of closing the entire hole and its accurate placement are required. Accurately obtaining this information by observing the surgical field from the anterior side and placing the buckling material in the correct position are highly challenging and require advanced knowledge and experience. Even with skilled surgeons, inaccurate placement of the buckling material can cause retinal redetachment [14]. In recent years, there has been a growing concern about an increase in the number of surgeons who have insufficient experience in buckling surgery; this has made it more challenging to determine the position of the buckling material by observing the surgical field from the anterior side by a surgeon.

In this study, we developed a standardized buckling technique that we propose for addressing this issue. The novelty of our technique lies in the utilization of the characteristics of anatomical positional relationships between the scleral insertion of the lateral rectus muscle, the vitreous base, and the ora serrata in determining the placement position of the buckle material. As mentioned earlier, particularly in cases of flap tear retinal detachment, it is important to determine whether the buckle is located around the peripheral side of the break. Depending on the extent of posterior vitreous detachment, the flap tear can extend from the equatorial region to the posterior margin of the vitreous base [15]. Conversely, if the buckling material can be placed from the posterior edge of the break to the posterior margin of the vitreous base, including the ora serrata, vitreous traction relief and temporary closure of the causative break can be achieved in most cases. The position of the scleral insertion of the lateral rectus muscle has been reported to overlap with the vitreous base insertion and ora serrata, allowing a rough estimation of the position of the ora serrata from external eye findings [16]. The reported distance from the ora serrata to the equator varies; it is generally approximately 5.5 mm [16–18]. Additionally, mattress sutures for buckling are usually placed with a 2-mm margin around the torn area, ensuring that the entire tear rests on the buckling protrusion. In other words, if a mattress suture with a width of 9–10 mm can be consistently placed relative to the ora serrata, in almost all cases, the buckling material can be placed in a position where relief and temporary closure of vitreous traction can be achieved without precise determination of the location of the causative retinal breaks during surgery. The #287 silicone tire used in this study had an appropriate mattress suture width of 9–10 mm [19], which satisfied the aforementioned requirements, and we believe that initial retinal reattachment was achieved in many cases. Therefore, this technique can estimate the position of the buckling material from external eye findings and can be performed solely as a standardized procedure regardless of the surgeon’s experience. Consequently, it is expected to markedly contribute to the homogenization of surgical outcomes.

In this study, there was a possibility of including cases where treatment could have been feasible with a smaller buckling material. However, considering the current situation in which the number of buckling surgeries and experienced surgeons for the treatment of RRD using buckling surgery is decreasing despite the unchanging importance of buckling surgery, this technique enables beginners to determine the position of the buckling material, regardless of the surgeon’s experience. This approach may aid in performing safer and more accurate surgeries and could be one of the solutions to the current challenges associated with buckling surgeries.

In this study, circumferential buckling was used in all cases. Therefore, one of the challenges was postoperative refractive changes. Circumferential buckling increases the axial length and induces myopia. Albanese et al [20] reported that segmental scleral buckling for RRD induced an increase in axial length and caused myopia, although these changes in the refractive index were significantly less than those observed with encircling elements. Wong et al [21] reported that the extent of segmental buckling was associated with an increase in postoperative axial length and myopia. Further research is required to understand the long-term outcomes and implications of these changes. In the future, it will be necessary to investigate whether such issues can be overcome by performing segmental buckling using the same buckling material (#287 silicone tires).

The limitations of this study include its retrospective nature, small sample size, lack of a comparative study design, and relatively short follow-up period. In the future, it will be desirable to increase the number of cases and to conduct prospective comparative studies. However, the standardized buckling surgery devised in this study is useful for retinal reattachment and may contribute to improved postoperative outcomes. Thus, this report is considered highly beneficial.

## Conclusion

We report the usefulness of standardized buckling surgery for primary RRD. This technique represents a novel approach that allows for a more standardized buckling procedure with less reliance on the surgeon’s experience, and it has the potential to contribute to improved outcomes in buckling surgery.

## Supporting information

S1 Video Overview of the novel surgical technique.
(MP4)
